# Artificial neural network trained on smartphone behavior can trace epileptiform activity in epilepsy

**DOI:** 10.1016/j.isci.2021.102538

**Published:** 2021-05-13

**Authors:** Robert B. Duckrow, Enea Ceolini, Hitten P. Zaveri, Cornell Brooks, Arko Ghosh

**Affiliations:** 1 Department of Neurology, Yale University, New Haven, CT, USA; 2 QuantActions GmbH, Lausanne, Switzerland; 3 Institute of Psychology, Leiden University, Wassenaarseweg 52, Leiden 2333 AK, The Netherlands; 4 Amherst College, Amherst, MA, USA

**Keywords:** Behavioral neuroscience, Bioengineering, Neural networks;

## Abstract

A range of abnormal electrical activity patterns termed epileptiform discharges can occur in the brains of persons with epilepsy. These epileptiform discharges can be monitored and recorded with implanted devices that deliver therapeutic neurostimulation. These continuous recordings provide an opportunity to study the behavioral correlates of epileptiform discharges as the patients go about their daily lives. Here, we captured the smartphone touchscreen interactions in eight patients in conjunction with electrographic recordings (accumulating 35,714 h) and by using an artificial neural network model addressed if the behavior reflected the epileptiform discharges. The personalized model outputs based on smartphone behavioral inputs corresponded well with the observed electrographic data (R: 0.2–0.6, median 0.4). The realistic reconstructions of epileptiform activity based on smartphone use demonstrate how day-to-day digital behavior may be converted to personalized markers of disease activity in epilepsy

## Highlights

• Brain implants can continuously log abnormal activity patterns in epilepsy.
• Real-world behavioral implications of the abnormal activity are not clear.
• An artificial neural network inferred brain activity based on smartphone behavior.
• Diurnal and other behavioral patterns were leveraged by the network.

## Graphical abstract

**Figure:**
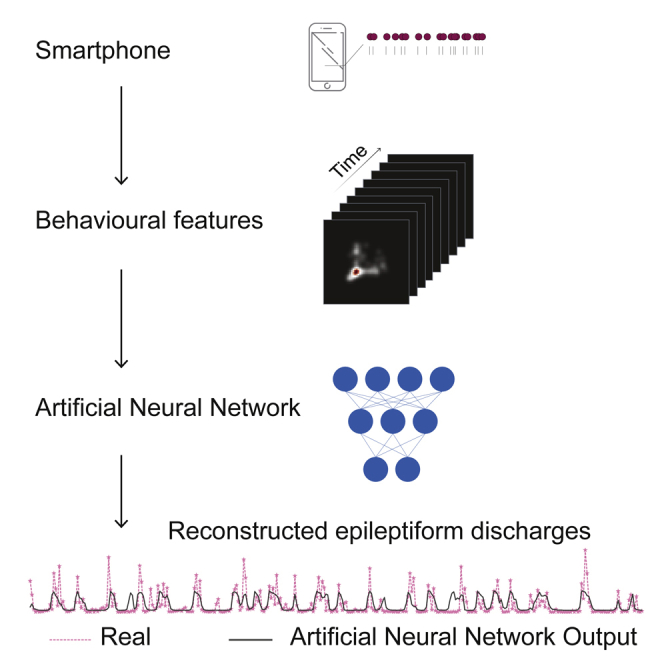

## Introduction

Epilepsy is defined by recurrent seizures driven by abnormal brain electrical activity (Engel and Pedley, 2008), as demonstrated by the electroencephalogram (EEG) during the seizure (Gibbs et al., 1937). Apart from ictal EEG activities, non-ictal but epileptiform EEG discharges can occur between seizures. As traditionally defined, these interictal discharges such as EEG spikes or spike and wave complexes are brief, usually lasting <400 ms. Although these discharges do not have behavioral consequences comparable to those accompanying clinical seizures, they are considered clinically useful, for instance, in localizing the irritative or seizure onset zones (de Curtis and Avanzini, 2001), and there is limited evidence that they relate to behavior and cognition (Faught et al., 2018; Helmstaedter et al., 2020; Kanner et al., 2020). Probing the behavioral correlates of epileptiform discharges is key toward unraveling their nature and clinical significance and may help address if the discharges should be treated (Fernández et al., 2015).

There are obstacles to establishing the behavioral correlates of epileptiform discharges. The discharges may be unique to each patient (Aanestad et al., 2020) and, as the timing of these discharges is not predictable, conventional behavioral assessments are near impossible. Also, any putative influence by what are essentially focal discharges on global function could have complex and difficult to quantify behavioral implications. Bounded by these limitations, there is scattered evidence that epileptiform discharges can imprint on memory tests or reaction times (Kleen et al., 2013; Matsumoto et al., 2013; Shewmon and Erwin, 1988). In one classic study of six patients, these barriers were overcome by continuously monitoring the driving of an instrumented vehicle in conjunction with the epileptiform discharges (Kasteleijn-Nolst Trenité et al., 1987). Interestingly, the discharges coincided with the inability to maintain course in two of the six patients and the inability to accurately report the odometer display in three. Such explorations during daily living conditions are rare (Faught et al., 2018).

To address the limitations of conventional cognitive testing, we used a continuously logged time series of day-to-day smartphone touchscreen events. In general, the inter-event probability distribution of human activities can be revealing of the underlying behavioral processes (Barabási, 2005; Malmgren et al., 2008, 2009). The probability distribution of smartphone touchscreen events too has been investigated to capture the putative behavioral processes (Pfister and Ghosh, 2020). In terms of cognitive processes, the fast touchscreen events captured by the probability distribution (25^th^ percentile) are correlated to indicators of executive functions as in motor variability and choice reaction times (Akeret et al., 2020; Balerna and Ghosh, 2018). These fast events also display substantial diurnal and weekly fluctuations (Huber and Ghosh, 2021). The other extreme of the inter-event probability distribution—the long gaps in smartphone usage—is indicative of sleep (Borger et al., 2019).

In this study, our measurements were conducted in a limited number of patients with epilepsy being treated with a responsive neurostimulator (RNS) system from NeuroPace, Inc. which is a closed-loop brain-responsive neurostimulation system with the ability to continuously monitor epileptiform discharges over years in a real-world environment (Baud et al., 2018; Duckrow and Tcheng, 2007; Kokkinos et al., 2019; Spencer et al., 2016). Importantly, these long-term recordings offer the necessary ground truth toward the discovery of correlative links between neural activity and peripheral measurements (Rao et al., 2021).

To establish neural-behavioral links, we correlated logs of smartphone touchscreen use with the occurrence of epileptiform discharges as recorded by the RNS System. We used a behavioral feature space based on joint interval distribution to capture the inter-touch intervals across timescales along with their short-term (next event) dynamics. Moreover, as social app usage distinctly correlates with brain measurements, we used an additional feature space generated by using inter-touch intervals gathered from apps such as Facebook or WhatsApp (Balerna and Ghosh, 2018; Westbrook et al., 2021). The artificial neural network model was trained to learn the correlations between epileptiform discharges and smartphone behavior in each subject. The neural network architecture used a combination of a state-of-the-art convolutional front end (He et al., 2016; Khan et al., 2020) to extract features from the behavior followed by a state-of-the-art recurrent neural network (RNN), using long short-term memory (Siami-Namini et al., 2018) to process the time-evolving behavioral features. After training, the model generated realistic reconstructions of epileptiform discharges based on smartphone behavior.

## Results

### Cyclical properties in epileptiform discharge counts and smartphone behavior

**Figure 1:**
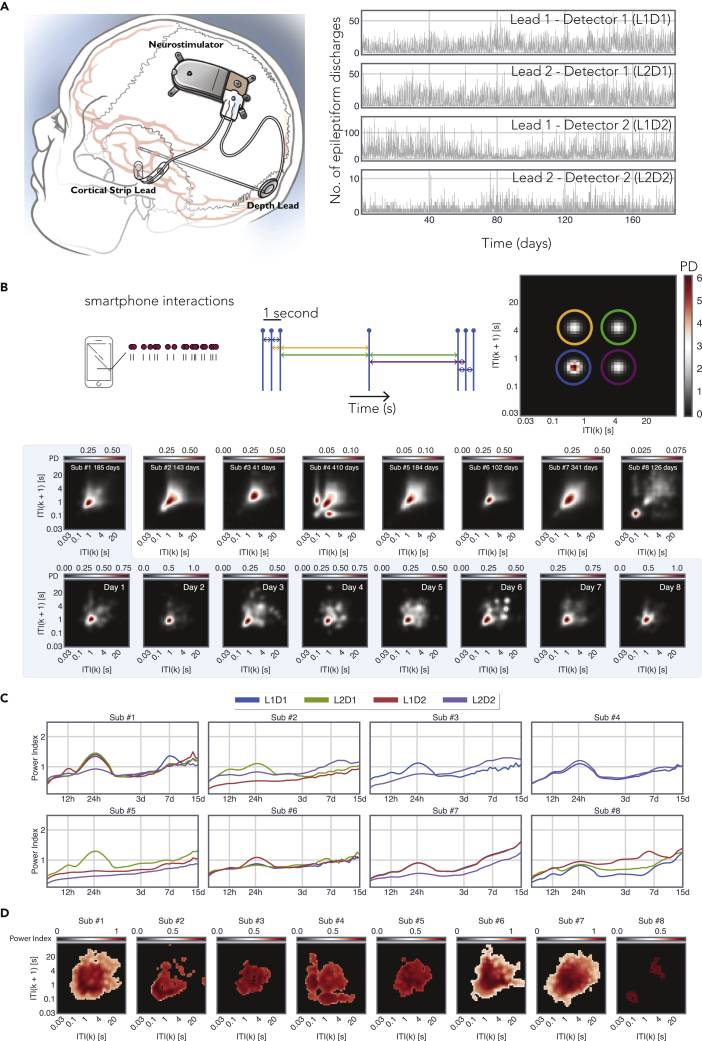
Combining long-term epileptiform records from an implanted device to smartphone behavioral data (A) Eight subjects were implanted with the RNS neurostimulator and NeuroPace cortical strip and depth leads (NeuroPace, Inc.) (see also Table S1). The location of the strip and the depth lead varied substantially from one subject to another, and the figure is for illustrative purposes. Detector activity from subject #1 was recorded for 185 days. The 4 leads tracked distinct activity patterns measured by two leads. (B) An illustration of the joint interval distribution that is used to capture the short-term behavioral dynamics on the smartphone by describing the relationship between an (say *k*) inter-touch interval (ITI) and the subsequent interval (*k*+1). Note that different parts of this feature space capture different forms of behavioral dynamics. For instance, the lower left corner represents rapid interactions and the diagonal represent rhythmic output where the subsequent intervals are the same. The probability distribution accumulated over the entire smartphone recording period is shown for each subject. The daily probability distributions are shown for subject #1. (C) The periodograms of the neural detector activity for each active detector from all the 8 patients used for this study. The periodograms were generated using the wavelet transform method described in (Baud et al., 2018). “L” corresponds to the lead number, and “D” corresponds to the detector number. (D) The power of 24 h cycles across the joint interval distribution. The non-significant power values according to shuffled bootstraps are masked (see also Videos S1, S2, S3, S4, S5, S6, S7, and S8 and Figure S1).

Hourly epileptiform discharge counts were gathered using the RNS System. There was substantial diversity in age, clinical reasons for the RNS System implantation, duration of the implant, and the location of the leads (see Table S1, for a clinical summary). The hourly counts of epileptiform discharges were not entirely random. The periodograms gathered from each active epileptiform detector revealed prominent peaks, albeit with substantial intra-individual and inter-individual variability (Figure 1). Notably, 24 h cycles were consistently seen across participants, but they too varied in their amplitude and width even within the same participant (i.e., across detector). Diurnal rhythms were also visible in the joint interval distributions derived from the subsequent smartphone inter-touch intervals (Figure 1). The regions of the smartphone behavioral space that showed cyclicity varied substantially from one person to the next. In addition to diurnal rhythms, we also observed multi-dien (21 days) cycles in smartphone behavior (Figure S1, for the day-to-day evolution in smartphone behavior, see Videos S1, S2, S3, S4, S5, S6, S7, and S8). Multi-dien cycles in the epileptiform discharges were occasionally observed—albeit less consistently than the 24 h cycle (Figure S2).

### Comparison of the model output to epileptiform discharge counts

**Figure 2:**
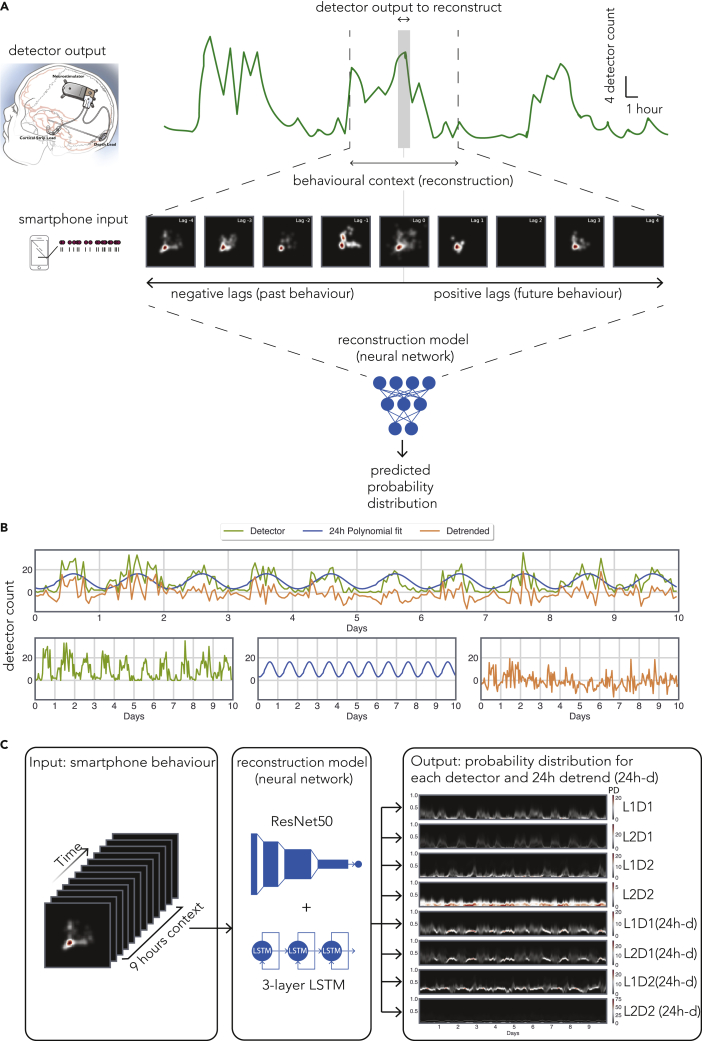
The features of the artificial neural network used to link smartphone behavior with epileptiform discharges (A) The overview of the model used to estimate the epileptiform discharge count from a given detector at a particular hour, *t* (shaded). The smartphone behavioral data spanning a 9 h window were used as the model input (*t*-4 h to *t*+4 h). The series of images represent hourly joint interval distributions introduced in Figure 1 with same scales. (B) A polynomial fit was used to detrend the epileptiform detector counts (see also Figures S2 and S3). (C) The model input consisted of the joint interval distribution of inter-touch intervals in general and inter-touch intervals based on social apps from the 9-h window. The model output consisted of the counts of the 4 detectors and the counts of the same detectors but detrended for 24 h cycles (labeled as “24 h-d”).

**Figure 3:**
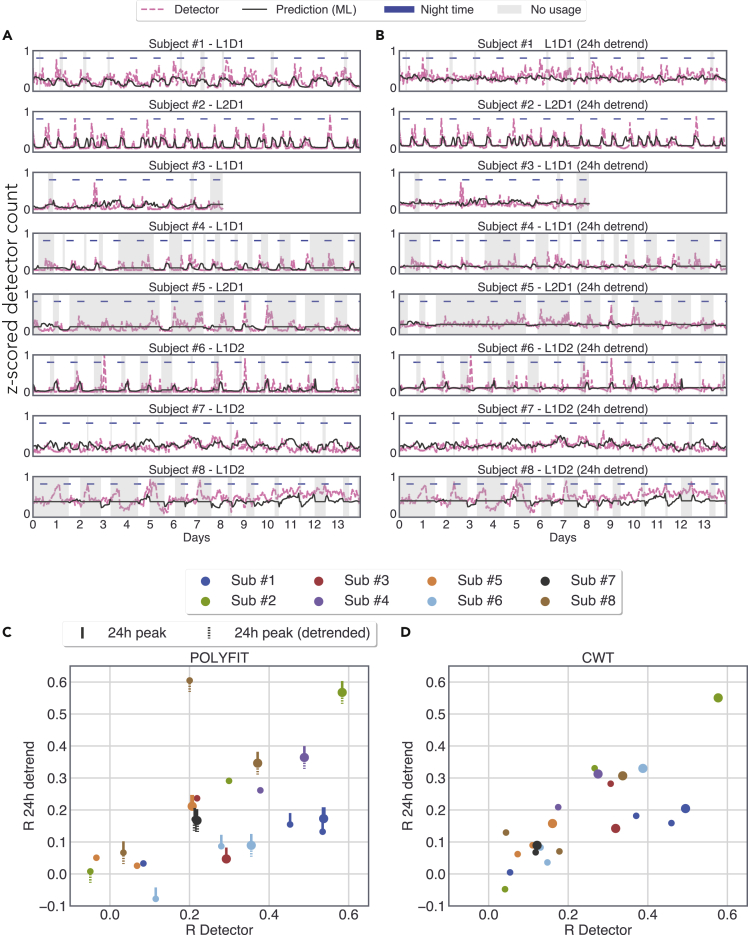
A personalized recurrent neural network model reconstructs realistic patterns of epileptiform discharges from smartphone behavior (A) The number of observed epileptiform discharges (detector hourly counts) and the model output based on smartphone touchscreen interactions. The model was trained on preceding data (smartphone interactions and epileptiform discharges) for each subject. The model used the behavioral data from the 9 h time bins surrounding each hour of the detector count. The shaded area depicts the periods where there was no smartphone data in any of the 9 h time bins. Note that the model outputs a mean value in the absence of smartphone data. The best performing detector from each subject according to R is shown (see also Figures S4–S8 and Table S2). (B) The number of discharges and the corresponding model output detrended of 24-h cycles using a polynomial fit. (C) The relationship between the R values obtained for the detector counts vs. the R values obtained for the 24-h detrended counts when using the polynomial method for detrending; upward tick indicates the presence of a significant 24-h cycle in the detector activity; downward dashed tick indicates the presence of a significant 24-h cycle in the detrended detector activity. (D) Same as (C) but using wavelet transformation for detrending 24-h cycles. In (B) and (C), big circles show the best performing detector for each subject according to R for the un-detrended activity; small circles of the same color show the remaining detectors.

The trained RNN model used the smartphone behavioral features as the input, and the model output was the predicted probability distribution of neural detector activity for all active detectors along with the 24 h detrended detector activity (Figure 2). Given the diverse nature of the 24 h peaks observed on the periodograms, we suspected the existence of both trivial 24 h cycles and non-trivial cycles containing multiple components. Therefore, we used two different strategies to detrend the 24 h cycles. First, we used a polynomial fit which preserved the rest of the frequencies and suppressed the trivial 24 h cycles (see periodograms in Figure S2). Second, we used a wavelet transformation to remove both trivial and non-trivial 24 h cycles (see periodograms in Figure S3). We obtained similar results in terms of model reconstructions and present the results from the polynomial fit here (Figure 3, for reconstructions based on the wavelet transformation, see Figure S4). For the best performing detectors in each patient, we found a strong relationship between the model output and the observed values. For subject #1, *R* = 0.53, *t*(789) = 17.88, p = 2.65 × 10^−60^; subject #2, *R* = 0.58, *t*(678) = 18.71, p = 2.46 × 10^−63^; subject #3, *R* = 0.29, *t*(172) = 4.01, p = 8.96 × 10^−5^; subject #4, *R* = 0.48, *t*(872) = 16.53, p = 1.23 × 10^−53^; subject #5, *R* = 0.21, *t*(222) = 3.14, p = 1.87 × 10^−3^; subject #6, *R* = 0.35, *t*(389) = 7.49, p = 4.56 × 10^−13^; subject #7, *R* = 0.22, *t*(1,283) = 8.03, p = 2.15 × 10^−15^; and subject #8, *R* = 0.37, *t*(171) = 5.22, p = 4.94 × 10^−7^. A similar pattern was visible for the 24-h detrended output with substantial loss for subject #3. For subject #1, *R* = 0.17, *t*(789) = 4.93, p = 9.79 × 10^−7^; subject #2, *R* = 0.56, *t*(678) = 17.95, p = 3.07 × 10^−59^; subject #3, *R* = 0.04, *t*(172) = 0.617, p = 0.53; subject #4, *R* = 0.36, *t*(872) = 11.54, p = 8.44 × 10^−29^; subject #5, *R* = 0.21, *t*(222) = 3.22, p = 1.44 × 10^−3^; subject #6, *R* = 0.09, *t*(389) = 1.77, p = 7.7 × 10^−2^; subject #7, *R* = 0.16, *t*(1,283) = 6.07, p = 1.66 × 10^−9^; and subject #8, *R* = 0.35, *t*(171) = 4.83, p = 3.03 × 10^−6^. For full reconstructions of the trimmed data displayed in Figures 3 and S4, see Figure S5, and for evaluations of the model output in terms of root mean squared error, see Table S2. We additionally estimated the model performance by comparing the model output with the real detector counts binned in 24-hr windows (Figure S6). In this reduced but diurnal rhythm free data, we found a significant correlation in 3 out of 8 individuals.

Given the substantial variation in model performance across detectors and patients, we addressed whether this variation could be explained by the two obvious candidates of (i) the sparseness of the detector activity or (ii) the density of the smartphone behavioral space (captured by its entropy). Although there was a linear relationship linking model performance and detector activity *R* = −0.5872, *t*(30) = −2.953, p =.008, the performance appeared unrelated to the density of smartphone behavior *R* = 0.1154 *t*(30) = 0.636, p = 0.529 (see Figure S7).

### Models based on behavioral data preceding the epileptiform discharges

When parallel RNNs were trained on the smartphone behavior preceding (9 h), the epileptiform discharge count bins, all subjects showed low to moderate though significant relationships between the model output and observed data (see Figure S8). For subject #1, *R* = 0.48, *t*(789) = 15.62, p = 3.67 × 10^−48^; subject #2, *R* = 0.30, *t*(687) = 8.32, p = 4.81 × 10^−16^; subject #3, *R* = 0.32, *t*(172) = 4.46, p = 1.46 × 10^−5^; subject #4, *R* = 0.38, *t*(872) = 12.07, p = 3.80 × 10^−31^; subject #5, *R* = 0.37, *t*(222) = 5.98, p = 8.58 × 10^−9^; subject #6, *R* = 0.40, *t*(389) = 8.71, p = 8.48 × 10^−17^; subject #7, *R* = 0.13, *t*(1,283) = 4.91, p = 1.02 × 10^−6^; and subject #8, *R* = 0.37, *t*(171) = 5.28, p = 3.91 × 10^−7^. A similar pattern was visible for the 24 h detrend output with a substantial loss of performance in subjects #1 & #3. For subject #1, *R* = −0.02, *t*(789) = −0.83, p = 0.40; subject #2, *R* = 0.29, *t*(687) = 7.99, p = 5.61 × 10^−15^; subject #3, *R* = 0.09, *t*(172) = 1.18, p = 0.23; subject #4, *R* = 0.24, *t*(872) = 7.55, p = 1.08 × 10^−13^; subject #5, *R* = 0.32, *t*(222) = 5.08, p = 7.70 × 10^−7^; subject #6, *R* = 0.39, *t*(389) = 8.60, p = 1.93 × 10^−16^; subject #7, *R* = 0.12, *t*(1,283) = 4.22, p = 2.63 × 10^−5^; and subject #8, *R* = 0.34, *t*(171) = 4.81, p = 3.34 × 10^−6^.

### Identifying the role of distinct smartphone behaviors on the model output

**Figure 4:**
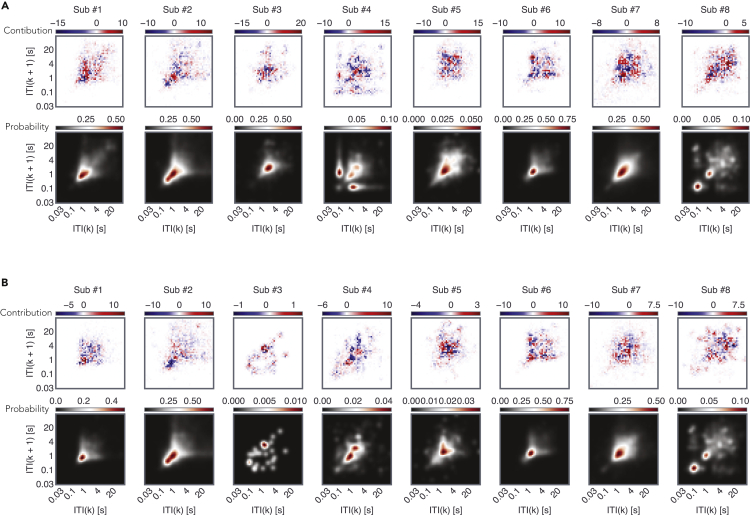
The diverse contributions of the different regions of the smartphone behavioral space to the model output Contributions were estimated using the gradient ∗ input method (see [Ancona et al., 2018]) to reveal the parts of the behavioral space that contributed to the rise in the model output (in red) and fall of the output (in blue). (A) The average contributions across the 9 hours bins used as input for the feature space consisting of all inter-touch intervals (see also Figure S9). (B) Same as (A) but for the feature space consisting of inter-touch intervals on social apps.

Using the model gradients, we addressed how different behavioral features were linked to the epileptiform discharges (Figure 4). One striking outcome of this analysis was that much of the behavioral space contributed to the model output. Essentially, the extreme gradients were not limited to only the fast inter-touch intervals. Second, the inter-individual differences were notable, indicating that the neuro-behavioral correlates were highly individualized. Third, the contributions of general inter-touch intervals were distinct from the inter-touch intervals selected from social apps. Finally, this analysis revealed that behaviors with similar dynamics could have a fundamentally different relationship to the discharge counts. For instance, for patient #2, inter-events under 1 s were associated with the falling part of the model output, whereas inter-events above 1 s were associated with the rising part. A similar pattern was of observations also evident for the time-causal model (see Figure S9).

## Discussion

The artificial neural networks learned the neuro-behavioral relationships such that epileptiform discharge hourly counts could be inferred from smartphone behavior—albeit with varying levels of success. These learned relationships support the existence of behavioral correlates of predominately subclinical epileptiform discharges.

The behavioral correlates of predominately subclinical epileptiform discharges were anticipated to be complex. The use of an RNN helped overcome the limitations of simple linear regression when used in the search of such correlations. We used the latest developments in artificial neural network analysis to establish how the behavioral feature space contributed to the model output (Ancona et al., 2018; Shrikumar et al., 2019). This analysis revealed a complex pattern of behavioral contributions to the artificial neural network output. Importantly, the model relied upon a broad array of behavioral features, and this emphasizes the need to use rich behavioral analysis in the search for neuro-behavioral correlates over the use of highly reduced behavioral space (for instance, extracting only the fastest 25^th^ percentile of inter touch intervals).

There were substantial inter-individual differences in model performance. These differences may stem from multiple factors such as the location of the irritative zone, the location of the electrodes, and the distinct electrophysiological properties of the epileptiform discharges. Nevertheless, our follow-up analysis did point to the more pragmatic contribution of data density—essentially, the sparser the detector activity, the worse the model performance. It now remains to be tested whether with longer durations of recording this limiting factor can be overcome.

We detected diurnal rhythmicity in the epileptiform discharge counts. This is in line with previous observations using similar long-term epileptiform discharge logs in RNS System-implanted patients (Baud et al., 2018; Duckrow and Tcheng, 2007; Spencer et al., 2016). To address whether this rhythm alone contributed to the model performance, we attempted to remove this 24-h cycle by using two different methods—but each method had its limitations. The simplistic polynomial fit was not well suited to the putatively compounded (complex) cycles and left some of the diurnal components intact and the wavelet-based method distorted the non-24 h signal components. Nevertheless, by examining the daily model outputs (binned by using a 24-h window), we established that, in 3 patients, the non-diurnal fluctuations present in the data were well captured by the RNN.

The RNN presented here established a correlative relationship between the smartphone behavior surrounding epileptiform discharges and the frequency of those discharges. To address whether the behavior preceding the discharges was related to that activity and address the possible effect of therapeutic stimulation, we trained a “time-causal” model using the smartphone behavior preceding the discharges. This model performed moderately well—suggesting that the behavior conducted in the hours before the epileptiform discharges contains information on future events. The finding that interictal epileptiform activity oscillates with subject-specific multi-day periods and that seizures occur preferentially during the rising phase of multi-day rhythms prompts the search for biomarkers of this activity (Baud et al., 2018; Karoly et al., 2021; Rao et al., 2021). Our findings support the possibility that epileptiform discharges too are preceded by behavioral precursors that could serve as such a biomarker for forecasting disease activity. However, the best performing forecast in our study had an R of 0.5, and we anticipate that the incorporation of other peripheral non-invasive measures may improve forecasting accuracy.

Improved reconstruction and forecasting models for epileptiform discharges may also help create closed-loop systems where therapeutic stimulations are based on not only the electrophysiological patterns but also—and perhaps more importantly—their real-world behavioral relevance. These improvements will be likely aided by the inclusion of discharge patterns along with discharge counts and the availability of recordings with a higher temporal resolution than that currently used (1 h).

### Limitations of the study

This study focused on a few individuals treated with the RNS System. The extent to which these findings can be generalized to the remaining RNS System-treated population or the more general population of persons with epilepsy remains to be seen. However, the inter-individual differences revealed here suggest that broad generalizations may not only be difficult but also be unnecessary—as the correlations can be drawn based on longitudinal measures at the level of each patient. Moreover, this study only partly addressed what underlay the model performance. Diurnal rhythm present in both the smartphone behavior and epileptiform discharges may contribute to the performance of the RNN. However, in select detectors without diurnal rhythms, the model performed moderately well, and in 3 individuals, the model output at a lower (daily) resolution corresponded well to the real discharge counts. These findings indicate that the behavioral correlates of epileptiform discharges are not just diurnal. Finally, the detector activity traced here triggered therapeutic stimulation, and whether the behavioral correlates of abnormal activity can be attributed to the intrinsic neuronal population activity or the subsequent stimulation remains unclear. Still, our findings based on the hours preceding the discharges suggest that at least some of the correlates can be attributed to intrinsic neural activity, and this is an encouraging step toward forecasting seizures (Kuhlmann et al., 2018; Nehlig and Wiebe, 2021).

## STAR★Methods

### Key resources table

**Table:** 

REAGENT or RESOURCE	SOURCE	IDENTIFIER
**Software and algorithms**		

Python version 3.8	Python Software Foundation	https://www.python.org
Tensorflow v2.2.0	Google	https://www.tensorflow.org/
KernelDensity from sklearn v0.24.1	Pedregosa et al. 2011	https://scikit-learn.org/stable/
Data processing, neural network model definition and training, statistical analysis	This paper	https://github.com/CODELABLEIDEN/iScience_ANN_RNS_Smartphone_2021
Smartphone touchscreen interaction logger	QuantActions Ltd	https://play.google.com/store/apps/details?id=com.quantactions.tapcounter

**Other**		

RNS system	Neuropace	https://www.neuropace.com/

### Resource availability

#### Lead contact

Further information and requests for code and data should be directed to and will be fulfilled by the lead contact Arko Ghosh (a.ghosh@fsw.leidenuniv.nl)

#### Materials availability

This study did not generate any materials.

#### Data and code availability

The code used to generate the results published in this paper can be accessed at https://github.com/CODELABLEIDEN/iScience_ANN_RNS_Smartphone_2021.

The dataset of brain activity and associated smartphone usage used in this publication is not made publicly available due to privacy related agreements but can be requested from the lead contact.

### Experimental model and subject details

#### Participants

We recruited 8 patients previously implanted with the RNS System for clinical indications, who also used a smartphone with the Android operating system. The subjects (4 female) ranged in age from 26 to 62 years (median 33) and had been implanted with the RNS System from 0 to 9.7 years (median 2.0) at the time the study began. Reasons for implantation included seizure onset in language cortex in 3 subjects, bilateral onset in 3 subjects, patient choice in 1 subject and rapid spread in 1 subject (Andrews et al., 2019). Regions probed by the RNS System included bilateral hippocampi in subjects with bilateral onset; temporal and frontal neocortex (2 left, 1 right) in subjects with language overlap; right mid- and anterior insula in the subject driven by choice; and left amygdala and anterior thalamus in the subject with a rapid spread. The clinical summary of the subjects is available in the data deposit (dataverse.nl, see Data Availability, below).

#### Ethics

All subjects gave written informed consent to the local Institutional Review Board-approved study (Yale School of Medicine Human Investigation Committee). The background smartphone App required the user to actively grant the necessary permissions and the associated privacy policy was available at all times. The data was coded and anonymized before analysis.

### Method details

#### RNS system and detection of epileptiform discharges

As part of standard clinical care, two RNS System (NeuroPace, Inc., Mountain View, CA, U.S.A.) leads were targeted to the presumed seizure-onset network to detect seizure onset and provide responsive electrical stimulation to interrupt the development of individual seizures (Lesser et al., 1999; Morrell, 2011) or modulate presumed seizure networks (Kokkinos et al., 2019). Strip leads with 10 mm contact spacing targeted cortical surfaces and depth leads with either 3.5 or 10 mm contact spacing targeted deep structures. Each lead had 4 contacts and consecutive independent pairs of contacts were used to measure bipolar signals. Treating physicians chose 2 of the 4 available intracranial EEG (icEEG) channels and could apply 2 user-configurable pattern detection algorithms independently to each bipolar channel. This resulted in 4 possible detector-channel pairs. These algorithms were tuned to detect the earliest electrographic signature of discharges that accompanied clinically reported seizures. After therapy is enabled, each detection triggers a responsive stimulation as a burst of biphasic square pulses with user-configured amplitude, frequency, pulse width and duration. Two bursts are delivered with each therapy and up to 5 therapies are delivered at ~1 s intervals between therapies as long as the icEEG signal meets detection criteria (Kokkinos et al., 2019; Morrell, 2011). Typical stimulation parameters for the subjects of this report were 100 ms bursts of 160 μs/phase pulses at 200 Hz. Amplitude varied from 1 to 10 mA (0.5 to 5.1 μC/cm^2^) but was constant for each subject.

Detection settings are tuned by clinicians to capture the onset of electrographic seizures. However, brief (~400 to 1000 ms) interictal epileptiform discharges, which are much more frequent, also trigger detections and subsequent stimulation. These epileptiform patterns include spike-wave discharges and bursts of rhythmic alpha or beta frequencies or low-voltage fast activity. Less commonly, abnormal activity meeting detection criteria can persist for longer periods measured in tens of seconds. These longer events can represent periods of abundant interictal spiking or electrographic seizures, for examples see (Baud et al., 2018). Hourly counts of these detections and other system events are stored by the implanted RNS® Neurostimulator for 28 days. Our subjects typically interrogated their neurostimulator weekly, providing continuous detector counts with hourly resolution. Detected events are also logged with 0.5 s resolution. However, because of memory constraints, the continuity of this record was limited by the event frequency and the time between neurostimulator interrogations and was too sparse for analysis.

#### Smartphone behavioral recordings

Smartphone touchscreen events were captured using an application (App) that operated in the background (TapCounter, QuantActions GmbH, Lausanne). The subject downloaded the App to their smartphone using the internet (Google Play, Google, Mountain View, CA). The App gathered three key variables: the timestamp of the screen on/off events, the timestamp of the touchscreen interaction and the label of the app in use (for example, com.facebook.katana for Facebook). The App collected the timestamps with a standard deviation of 15 ms (Balerna and Ghosh, 2018). The apps were further categorized according to Social and non-Social based on the Google Play Store category. These data were downloaded from cloud-based services and parsed using scripts provided by QuantActions GmbH and implemented in MATLAB (Mathworks, Natick, MA).

#### Smartphone behavioral feature extraction

We captured short-term screen tapping dynamics using the joint interval distribution of subsequent inter-touch intervals (ITI). The ITIs occurring within a smartphone usage session, defined as the time between a screen-on event and a screen-off event, were considered further. Any ITI *K* was related against the subsequent *K*+1. The probabilities as shown in Equation 1,

$$P\left(log_{10}\left(x_{k+1}\right)\;,\;log_{10}\left(x_{k}\right)\right)$$

where x indicates the ITI value and its subscript indicates the time index, were estimated using kernel density estimation with the ITIs accumulated over 1 h. The window size of 1 h was selected to match the resolution of the neural detector records. The 2-dimensional kernel density estimation was done with a Gaussian kernel and a bandwidth of 0.1 (Pedregosa et al., 2011). The output of the kernel density estimation was then discretized using 50 bins per dimension in the range of 1.5 and 5 (corresponding to 30 ms – 100 s). The range was selected as the 0^th^ and 99^th^ percentile of all the ITIs across all the subjects. The Social app behavioral features were identically extracted, except that we selected ITIs based on the Social app category. The android system clock (time zone corrected) was used to align with the RNS System data. (Joint interval distribution can be obtained from a series of discrete timestamp with the function ` dt_to_JID` in the script `utils.py`)

#### Analysis of epileptiform and behavioral cycles

We characterized the nature of the detectors' activity following the analysis of ultradian, circadian and multidien cycles analysis as described in (Baud et al., 2018). In brief, the periodogram of each detector's activity was estimated using a complex continuous wavelet transform (see function `periodgram` in the script `utils.py`). The average periodograms (power spectra) were obtained as the square root of the average over time of the absolute value of the transform at each scale. Significance for the periodogram peaks was obtained via bootstrap by shuffling the time series 1000 times and estimating the corresponding 95% confidence intervals (see function `bootstrap_periodgram` in the script `utils.py`).

To extract the cycles in behavioral data, we performed the same analysis on the 2D behavioral joint interval distributions. In particular, we calculated the periodogram for each "pixel" in the 2D feature space. Significance for each pixel was obtained by calculating a 95% confidence interval from a surrogate time series obtained via bootstrap by shuffling the time series of each pixel separately 100 times (instead of 1000 due to computational constraints) as described for the epileptiform discharges. Significant clusters smaller than a square of 2 x 2 pixels were masked (see script `bootstrap.py`).

Two different methods were used to detrend the epileptiform discharge counts. The first method used a 24 h polynomial fit of degree 5 (function ` split_season` in script `utils.py`). The degree was selected from the range [2-10] as the degree that gave the best fitting performance using test data obtained from an 80/20 split across all subjects and detectors. The second detrending method removed the 24 h spectral components during reconstruction of the wavelet decomposed signal (function ` remove_24h_cwt` in `utils.py`). Although this removed the 24 h cycles it is noted that it also reduced the data further as only those signal features captured by wavelets in the first place contribute to the detrended re-composition.

#### Model architecture

The model’s objective was to estimate the value of all detector's activity at a given hour and the 24 h detrended value at the same hour. In subjects where all detectors were active, it consisted of 8 output channels (4 channels of epileptiform activity + 4 detrended epileptiform activity). The input to this model contained the hourly behavioral feature spaces based on all ITIs and Social app ITIs. The length of the behavioral sequence was set to 9 h based on cross-validation including minimizing overfitting.

In the model, the first feature extraction segment operated in parallel on the 2D joint interval distribution for each time step. The second segment processed the time series obtained from the features extracted by the first segment at each time step of the context. The feature extraction segment used a ResNet50 architecture which is commonly used in computer vision tasks (K. He et al., 2016). This segment was applied to all of the ‘context’ input (spanning 9 h) in parallel. The output from the last layer of this segment (1024-dimensional vector per time step) was fed into a 3-layer LSTM network (with 50 units per layer). Note, in general, LSTM layers improve artificial recurrent neural networks performance by leveraging complex sequential features (S. Siami-Namini et al., 2018). This layer output predictions after processing the 9 h time series (see the script `resnet_utils.py` for the details on model architecture).

To incorporate in the model the notion of uncertainty and avoid assuming the shape of the output conditional distribution P(detectors's activity | smartphone use), the model did not output a scalar but a mixture of 3 Gaussians (mixture density model)(Bishop, 1994). In particular, the model output consisted of 9 values corresponding to the 3 means, standard deviations and mixing coefficients for the 3 Gaussians in the mixture. The network was then trained to minimize a negative log-likelihood objective. For evaluation, we obtained a scalar estimation from each time step using Maximum likelihood (ML) on the output mixture of Gaussians (for details on how the model output is built and how the negative log-likelihood is calculated for training purposes see the script `MDN.py`). Given a large number of parameters in the model (6 Million) we used l2 regularization (lambda=0.001) on all the features maps of the ResNet50 feature extraction segment as well as batch normalization. We used a dropout (p=0.4) between the recurrent layers in the second segment and finally a l1 regularization (lambda=0.001) on the last layer that outputs the Gaussian mixture parameters. For each subject, we trained a different model using 80% of the available data for training and 20% of the *subsequent* data for testing. We trained for 100 epochs using Adam optimizer with a learning rate of 1 e^-3^. We evaluated the model in terms of Pearson's correlation coefficients, R (derived from periods where smartphone data was present in at least one of the 9 h bins) and RMSE between the true hourly count and the estimated hourly count. Note, of these two evaluations we report the R in the main text as RMSE is sensitive to signal range and the range variation from one detector to the next.

#### Measures of data channel activity

We measured the activity levels of the detector and 2D smartphone behavioral feature space using two different methods. For the 1D detector activity we used sparsity: a ratio of the number of hour bins with no detector's activity (hourly count = 0) vs. the total number of hours of recordings. A sparsity of 1 would mean the detector was never active, a sparsity of 0.0 would mean there was always at least 1 event per hour. For the 2D smartphone behavioral space we used entropy: defined as in equation 2:

$$-\int \int P\left(log_{10}(x_{k+1})\;,\;log_{10}\left(x_{k}\right)\right)log_{2}\left(P\left(log_{10}\left(x_{k+1}\right)\;,\;log_{10}\left(x_{k}\right)\right)\right)dx_{k}dx_{k+1}$$,

where x denotes ITI value and its subscript indicates the time index (see function `JID_entropy` in `utils.py`). The overall entropy for one subject was estimated as being the average entropy across hours.

### Quantification and statistical analysis

The statistics of R for each subject and each detector (with and without 24h detrending) were obtained with a two-tailed t-test with the null hypothesis that the coefficient is 0. We used the p-values for R obtained from the t-test described above and applied a Benjamini/Hochberg FDR correction for independent or positively correlated variables with a threshold set at 0.05. The statistical significance for 24 h peaks in Figure 3 was obtained by using bootstrap to create 1000 surrogate time series and by using the quantile of the obtained distribution to construct a 99.9 percent confidence interval. Similarly, the statistical significance for each JID pixel in Figure 1 was obtained with bootstrapping and construction of a 99.9 percent confidence interval.

## Supplemental information

Document S1. Figures S1–S9 and Tables S1 and S2

Video S1. Day-to-day fluctuations in smartphone behavior as captured using the joint interval distribution in patients #1, related to Figure 1

Video S2. Day-to-day fluctuations in smartphone behavior as captured using the joint interval distribution in patients #2, related to Figure 1

Video S3. Day-to-day fluctuations in smartphone behavior as captured using the joint interval distribution in patients #3, related to Figure 1

Video S4. Day-to-day fluctuations in smartphone behavior as captured using the joint interval distribution in patients #4, related to Figure 1

Video S5. Day-to-day fluctuations in smartphone behavior as captured using the joint interval distribution in patients #5, related to Figure 1

Video S6. Day-to-day fluctuations in smartphone behavior as captured using the joint interval distribution in patients #6, related to Figure 1

Video S7. Day-to-day fluctuations in smartphone behavior as captured using the joint interval distribution in patients #7, related to Figure 1

Video S8. Day-to-day fluctuations in smartphone behavior as captured using the joint interval distribution in patients #8, related to Figure 1
