# Supplementary material for: Artificial neural network trained on smartphone behavior can trace epileptiform activity in epilepsy
Source: iScience. 2021 May 13;24(6):102538. doi: 10.1016/j.isci.2021.102538 (PMC8257969; doi:10.1016/j.isci.2021.102538)
Supplement: Document S1. Figures S1–S9 and Tables S1 and S2 [file mmc1.pdf]

iScience, Volume *24*

Supplemental information

Artificial neural network trained on smartphone behavior can trace epileptiform activity in epilepsy

Robert B. Duckrow, Enea Ceolini, Hitten P. Zaveri, Cornell Brooks, and Arko Ghosh

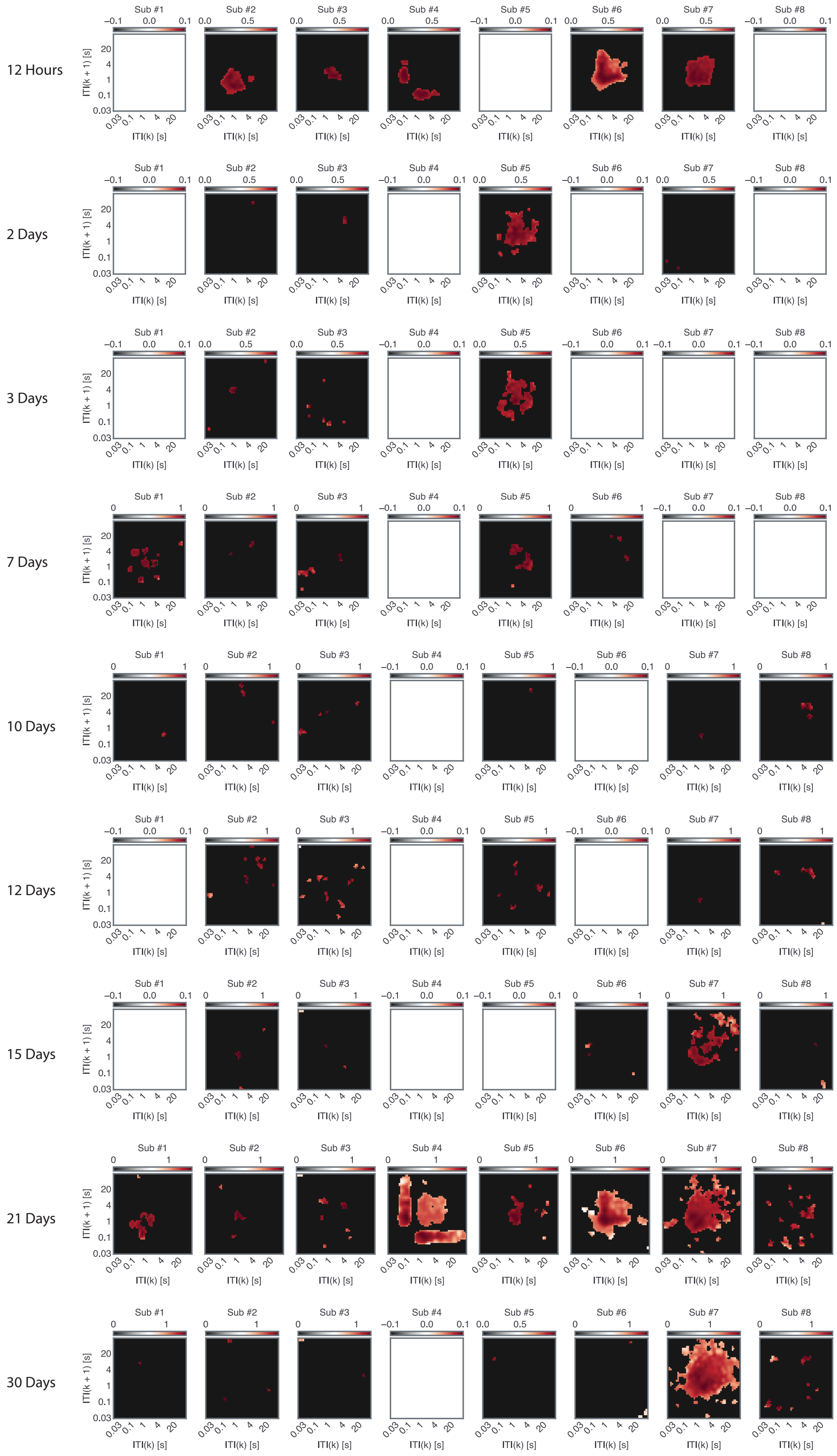

Figure S1. Additional analysis to measure 12 h and multidien rhythms in smartphone behavior, related to Figure 1.

Figure S1. The power of 12h, 3 d, 7d, 10 d, 12 d, 15 d, 21 d, 30 d cycles. The non-significant power values according to shuffled bootstraps are masked. These cycles were selected based on previously reported multidien cycles in epileptiform discharges. Note the sample durations reported in Table S1 before inferring the long-period cycles.

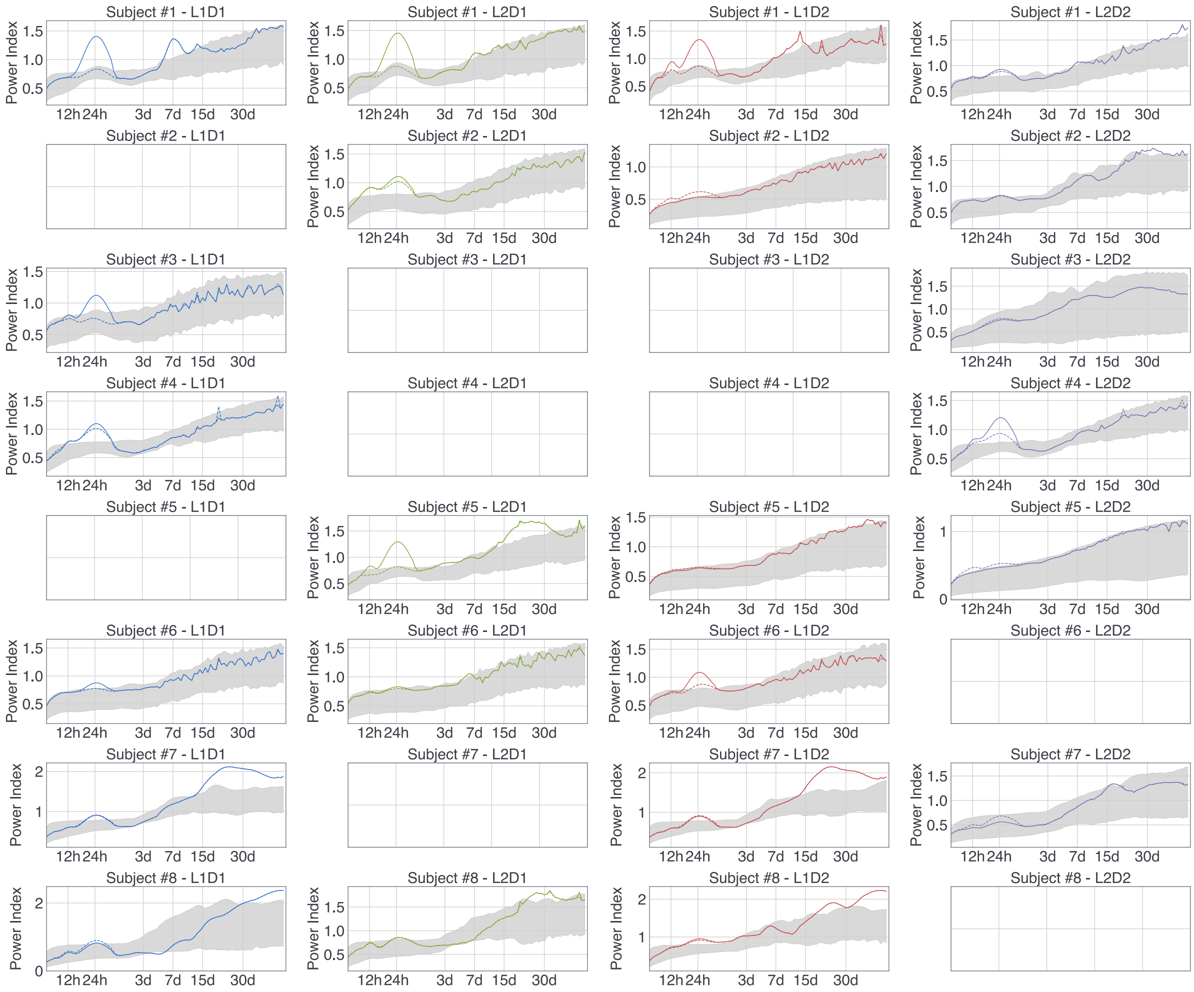

Figure S2. Detrending of 24 h cycles using polynomial fit, related to Figure 2.

Figure S2. The periodogram of each detector data (solid line) along with the distribution of values obtained from bootstrapped shuffled data (shaded area). The consequences of the polynomial fit detrending on the periodogram (dashed line). L corresponds to the lead number and D corresponds to the detector number. The rows correspond to patients #1 to #8.

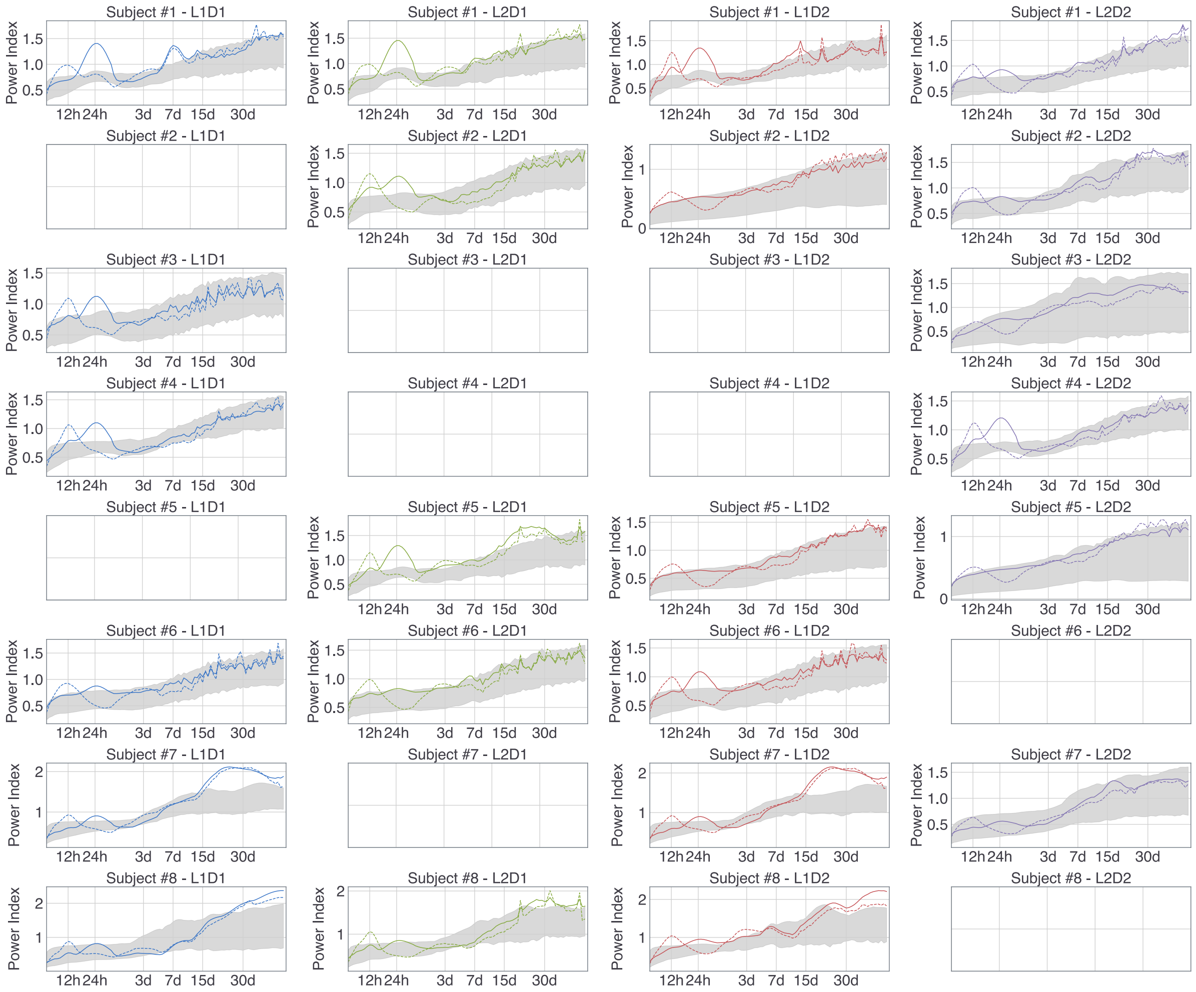

Figure S3. Detrending of 24 h cycles using wavelet transformation, related to Figure 2.

Figure S3. The periodogram of each detector data (solid line) along with the distribution of values obtained from bootstrapped shuffled data (shaded area). The consequences of the wavelet transform detrending on the periodogram (dashed line). L corresponds to the lead number and D corresponds to the detector number. The rows correspond to patients #1 to #8.

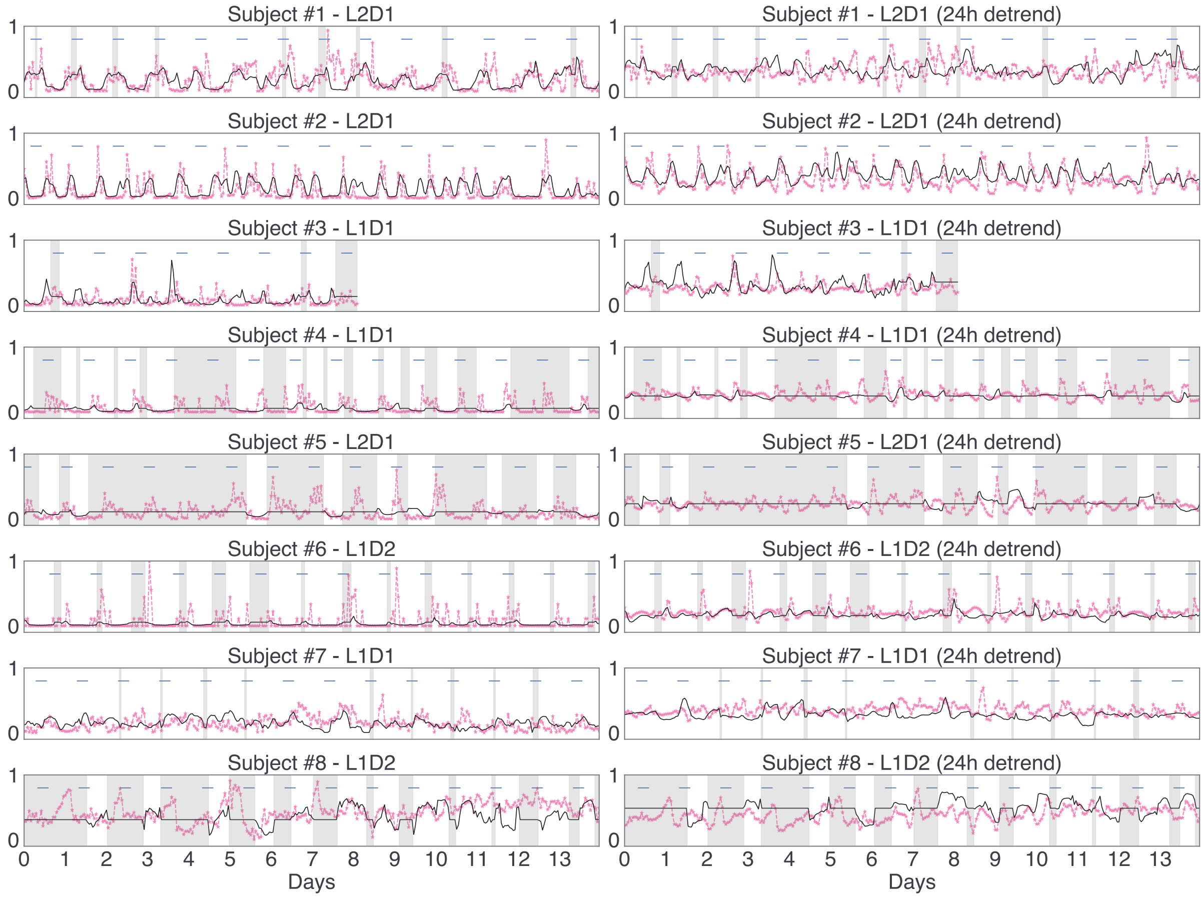

Figure S4. Reconstruction of discharge counts based on wavelet transformed detrending of 24 h cycles, related to Figure 3.

Figure S4. The legend follows the one in Figure 3 panels (a, b).

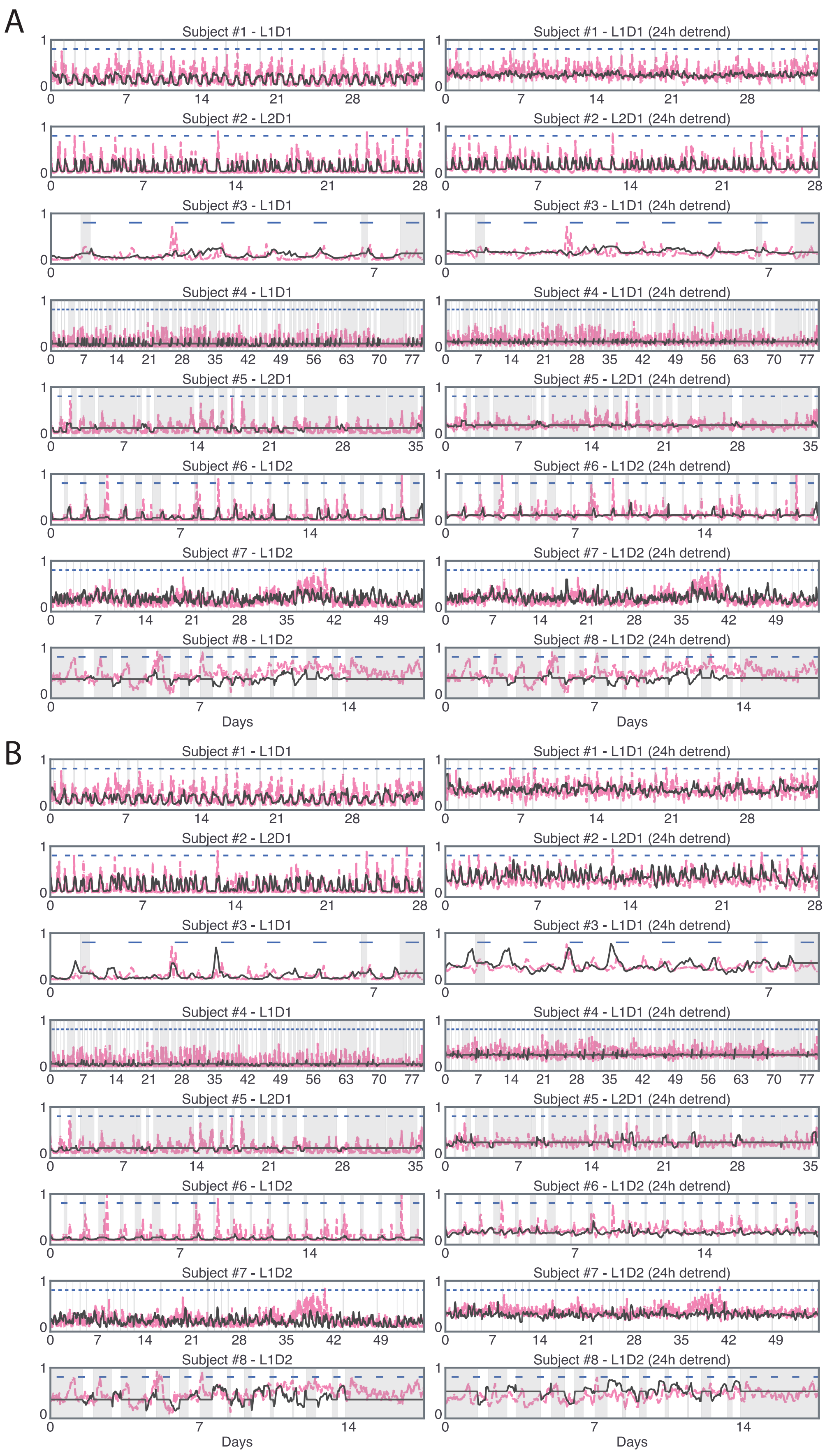

Figure S5. Full traces of model prediction of discharge counts, related to Figure 3.

Figure S5. The model prediction of discharge counts for reconstruction with (A) polynomial fit detrending, (B) reconstruction with cwt-based detrending.

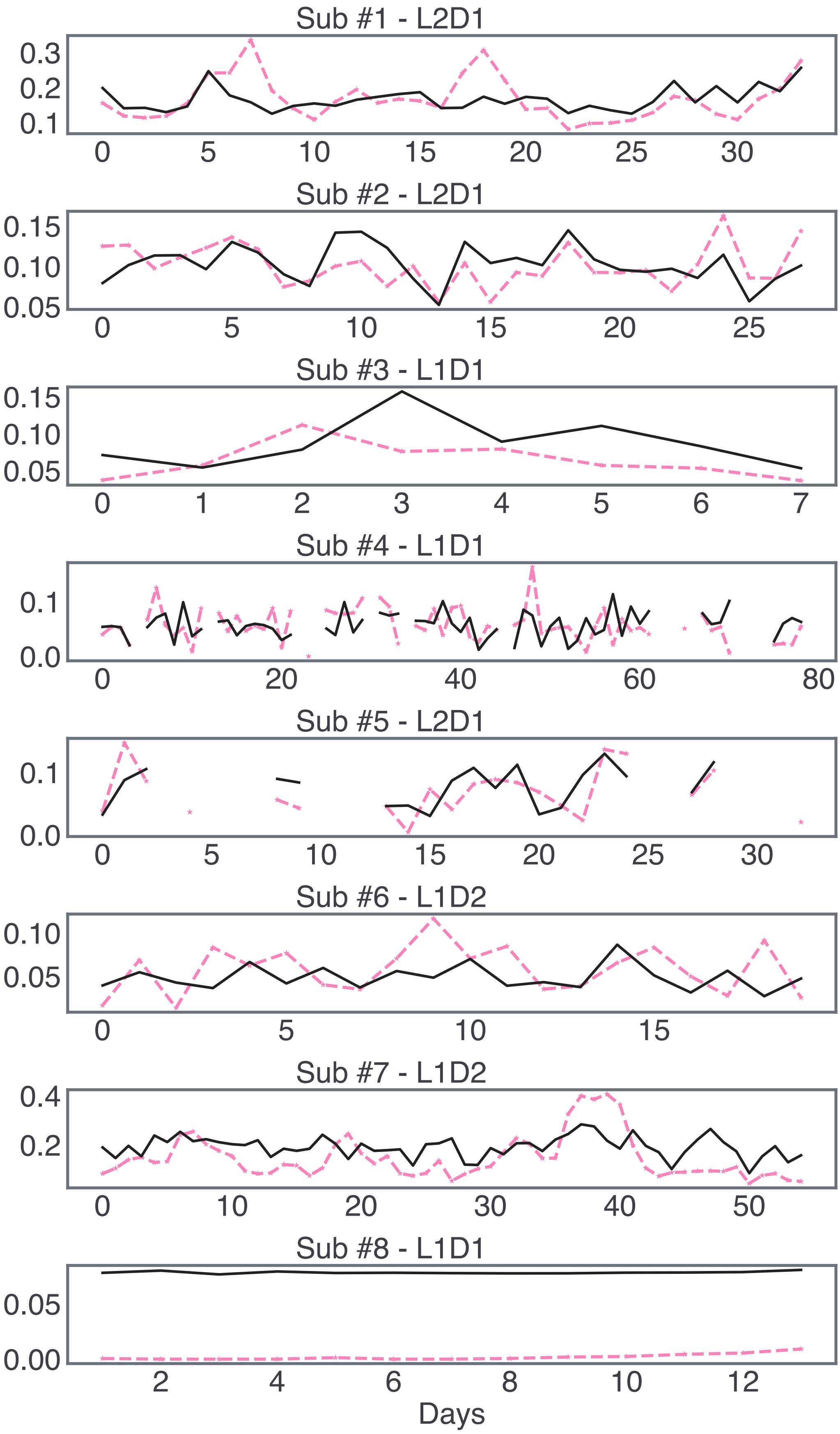

Figure S6. Model prediction of discharge counts for reconstruction binned in 24 hours windows, related to Figure 3.

Figure S6. Only the best detector is shown for each subject. The legend is the same as in Figure 3 (a). After false discovery rate correction we found significant correlations in 3 of the 8 subjects. For subject #1, $R = 0.42$, $t(32) = 2.65$, $p = 3.11 \times 10^{-2}$; subject #5, $R = 0.57$, $t(19) = 3.09$, $p = 1.60 \times 10^{-2}$; subject #7, $R = 0.51$, $t(53) = 4.27$, $p = 2.52 \times 10^{-4}$.

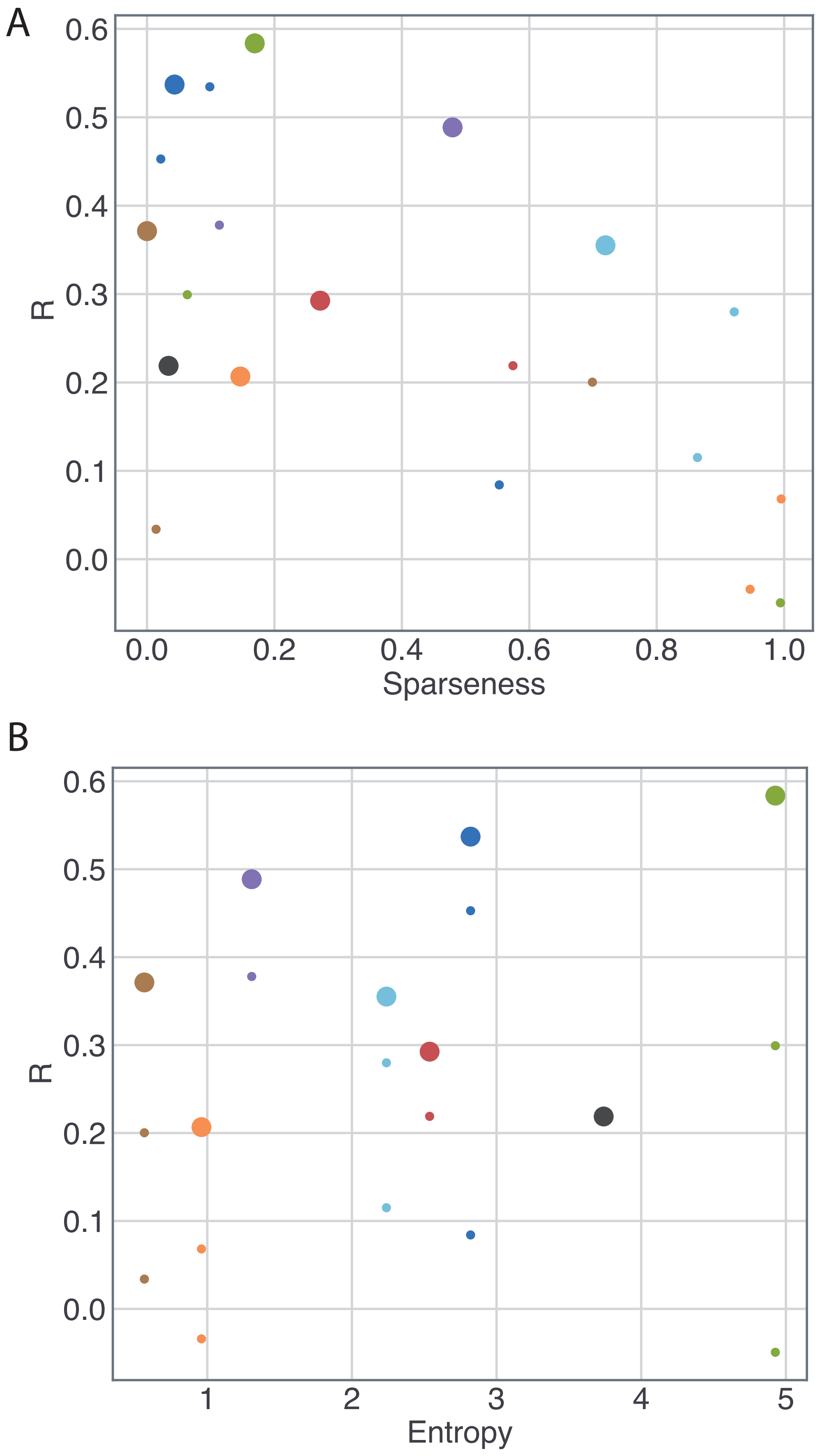

Figure S7. Inter-individual differences in model performance, related to Figure 3.

Figure S7. (A) The relationship between the sparsity of the detector counts and the performance of the model to reconstruct discharge counts. (B) The relationship between the smartphone behavioral entropy (to capture the corresponding measurement density) and the performance of the model to reconstruct discharge counts.

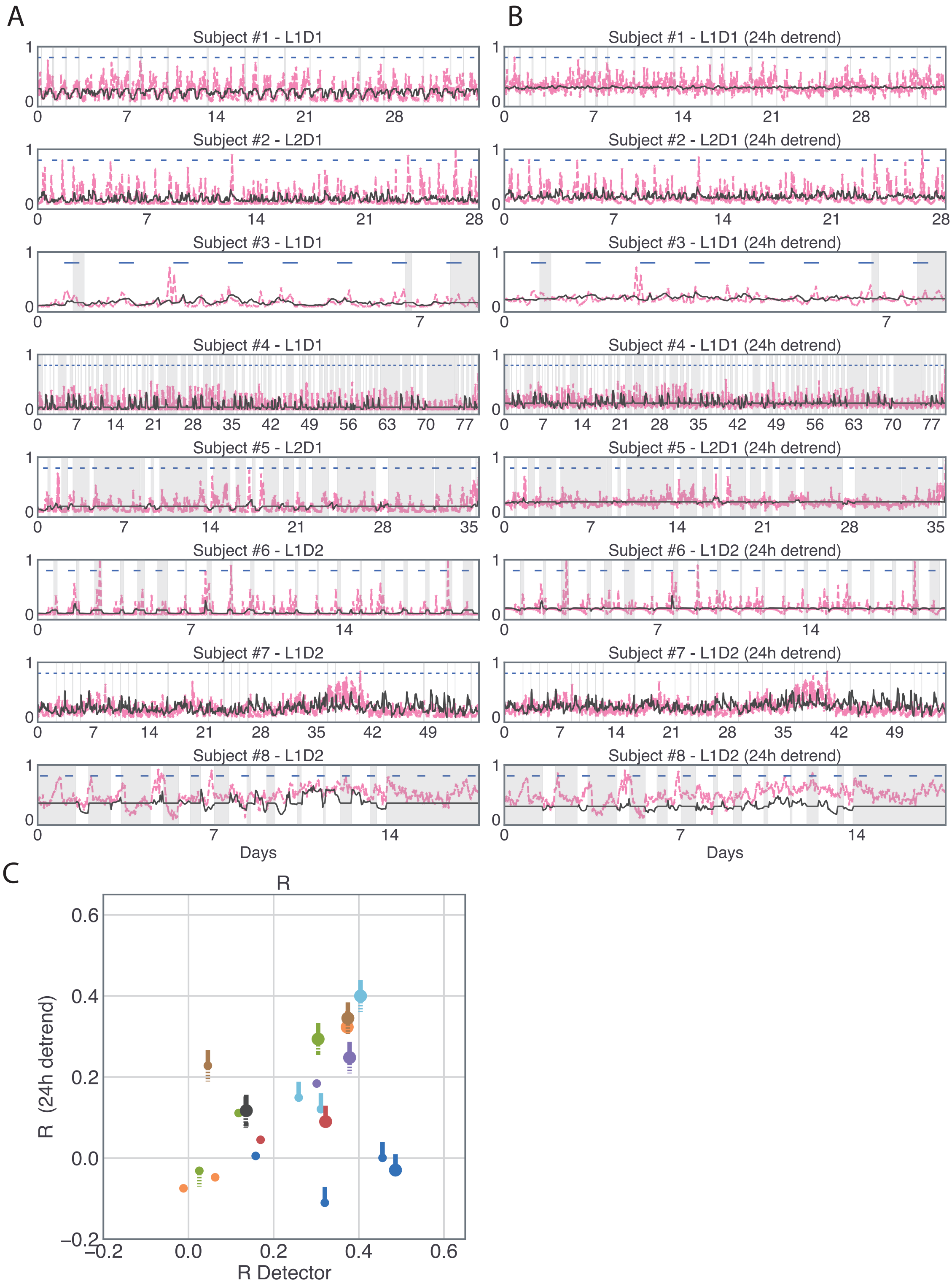

Figure S8. Forecasting of discharge counts based on 9 hours of smartphone data preceding the discharge counts, related to Figure 3.

Figure S8. The legend is the same as in Figure 3 (A, B, C) except that a ‘time-causal’ model was used.

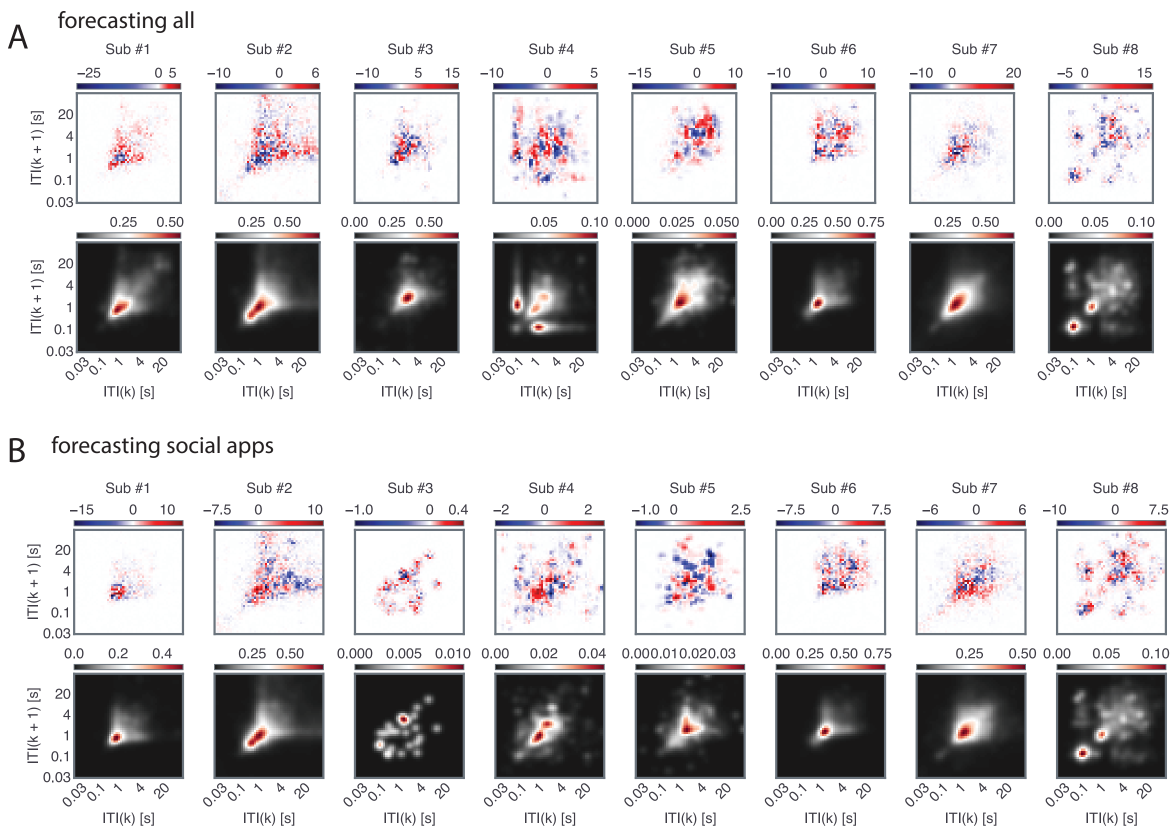

Figure S9. The diverse contributions of the different regions of the smartphone behavioral space to the causal model output, related to Figure 4.

Figure S9. Same as figure 4 but for forecasting/time-causal model.

ID	Age at Study (≈ 5 yrs)	MRI findings	Reason for RNS System	RNS System Lead 1	RNS System Lead 2	Smartphone Touches (median/day)	Years Implanted	Analysis epoch (days)
1	25	No lesions	bitemporal onset	R hippocampus	L hippocampus	1002.00	4.6	139
2	30	No lesions	language overlap	R superior temporal gyrus	R inferior frontal gyrus	2769.00	1.1	142
3	60	L FT encephalomalacia	bitemporal onset	L hippocampus	R hippocampus	408.00	0.2	41
4	25	hippocampal loss of architecture	language overlap	L orbital frontal gyrus	L orbital frontal gyrus	859.00	3.3	594
5	30	L anteror mesial encephalomalacia	language overlap	L middle frontal gyrus	L superior temporal gyrus	313.00	2.2	183
6	60	nodular heterotopia	bitemporal onset	L hippocampus	R hippocampus	2369.00	10.4	369
7	35	R TO dysplasia	patient choice over LITT	R insula - middle	R insula - anterior	5976.00	1.2	244
8	55	L amygdala enlargement	rapid spread	L amygdala	L thalamus - anterior nucleus	102.00	0.5	37

Abbreviations: L, left; R, right; F, frontal; T, temporal; O,

Table S1. Clinical summary of the patients used in this study, related to Figure 1.

ID			1	2	3	4	5	6	7	8
Reconstruction	Full	R	0.53	0.58	0.29	0.49	0.21	0.35	0.22	0.37
		RMSE	8.68	20.34	5.05	8.13	8.40	1.16	13.61	35.52
	24h detrend	R	0.17	0.56	0.04	0.36	0.21	0.09	0.16	0.34
		RMSE	8.76	20.93	4.88	7.48	7.02	1.17	13.17	35.86
Forecasting	Full	R	0.48	0.30	0.32	0.38	0.37	0.40	0.14	0.37
		RMSE	8.63	23.87	4.76	10.03	10.93	0.84	13.39	41.58
	24h detrend	R	-0.02	0.29	0.09	0.24	0.32	0.39	0.11	0.34
		RMSE	8.24	24.21	4.56	9.52	9.29	0.77	13.50	55.14

Table S2. RMSE values juxtapositioned to the R values of the best performing detectors (original and 24-h de-trended) for each patient for both reconstruction and forecasting of epileptiform discharge counts, related to Figure 3.
